# Acceleration of Mesenchymal-to-Epithelial Transition (MET) during Direct Reprogramming Using Natural Compounds

**DOI:** 10.4014/jmb.2208.08042

**Published:** 2022-09-19

**Authors:** Ji-Hye Seo, Si Won Jang, Young-Joo Jeon, So Young Eun, Yean Ju Hong, Jeong Tae Do, Jung-il Chae, Hyun Woo Choi

**Affiliations:** 1 Department of Dental Pharmacology, School of Dentistry, Jeonbuk National University, Jeonju 54896, Republic of Korea; 2 Department of Agricultural Convergence Technology, Jeonbuk National University, Jeonju 54896, Republic of Korea; 3 Disease Target Structure Research Center, Korea Research Institute of Bioscience and Biotechnology (KRIBB), Daejeon 34141, Republic of Korea; 4 Musculoskeletal and Immune Disease Research Institute School of Medicine, Wonkwang University, Iksan 54538, Republic of Korea; 5 Department of Psychiatry and Molecular Neurobiology Laboratory, McLean Hospital and Program in Neuroscience, Harvard Medical School, Belmont, MA 02478, USA; 6 Department of Stem Cell and Regenerative Biotechnology, KU Institute of Science and Technology, Konkuk University, Seoul 05029, Republic of Korea; 7 Department of Animal Science, Jeonbuk National University, Jeonju 54896, Republic of Korea

**Keywords:** iPSC, LCD, reprogramming, in vitro, natural compound, MET

## Abstract

Induced pluripotent stem cells (iPSCs) can be generated from somatic cells using *Oct4*, *Sox2*, *Klf4*, and *c-Myc* (OSKM). Small molecules can enhance reprogramming. Licochalcone D (LCD), a flavonoid compound present mainly in the roots of *Glycyrrhiza inflata*, acts on known signaling pathways involved in transcriptional activity and signal transduction, including the PGC1-α and MAPK families. In this study, we demonstrated that LCD improved reprogramming efficiency. LCD-treated iPSCs (LCD-iPSCs) expressed pluripotency-related genes *Oct4*, *Sox2*, *Nanog*, and *Prdm14*. Moreover, LCD-iPSCs differentiated into all three germ layers in vitro and formed chimeras. The mesenchymal-to-epithelial transition (MET) is critical for somatic cell reprogramming. We found that the expression levels of mesenchymal genes (*Snail2* and *Twist*) decreased and those of epithelial genes (*DSP*, *Cldn3*, *Crb3*, and *Ocln*) dramatically increased in OR-MEF (OG2^+/+^/ROSA26^+/+^) cells treated with LCD for 3 days, indicating that MET effectively occurred in LCD-treated OR-MEF cells. Thus, LCD enhanced the generation of iPSCs from somatic cells by promoting MET at the early stages of reprogramming.

## Introduction

Somatic cells can be reprogrammed by enforcing ectopic transcription factors (*Oct4*, *Sox2*, *Klf4*, and *c-Myc*). However, induced pluripotent stem cells (iPSCs) are generated with low efficiency in vitro [1]. Despite steady progress in reprogramming studies, the molecular mechanisms that coordinate reprogramming transition are unclear [2]. Current reprogramming studies suggest that the reprogramming stages can generally be divided into early and late stages and are regulated by a specific transcriptional co-regulator [3-5]. Differentiated cells follow a similar sequence of epigenetic changes when somatic cells are reprogrammed, such as undergoing transcriptional and histone modification changes prior to DNA methylation changes [6]. Jose M. P. and colleagues showed that the expression of reprogramming-related genes was altered in reprogramming cells in two phases of iPSC formation. Refractory cells (Thy1-positive cells) in reprogramming cells contained fewer reprogramming factors (OKSM) than Thy1-negative cells, and degradation of reprogramming factors was detected by ubiquitination in Thy1-positive cells [7]. The mesenchymal-to-epithelial transition (MET) is an essential process in the early stages of somatic cell reprogramming into iPSCs [4]. The transcriptome and extracellular matrix are regulated in the early stage of reprogramming by MET, and the reprogrammed cell morphology changes while the proliferation rate increases [8]. A recent study showed that *Sox2*/*Oct4* and *c-Myc* regulate MET progression and suppress the expression of mesenchymal genes (Snail), and *Klf4* regulates the expression of epithelial genes [4]. Pluripotent reprogramming is an inefficient process due to the presence of various defined and unidentified barriers. Other recent studies have shown that small molecules can be used to improve iPSC induction by regulating DNA methylation and histone modification [9-11]. Moreover, a study reported that SB431542 is an inhibitor of TGF-β receptors and could regulate MET transition and enhance production of iPSCs [4, 12]. However, in the reprogramming fields, few natural compounds have been exploited [13, 14], and therefore, further research is needed to discover their untapped potential. Licochalcone D (LCD) is a natural compound found mainly in the roots of *Glycyrrhiza inflata* (licorice root), which is used in traditional medicine to treat bronchial asthma, gastric ulcers, and inflammation. The anti-inflammatory effect of *Glycyrrhiza inflata* has been demonstrated by its strong inhibition of NF-kB by LCD [15]. In addition, LCD was confirmed to be related to the inhibition of NF-kB, which plays an important role in the epithelial-mesenchymal transition (EMT) process [16].

We demonstrated that LCD improves reprogramming efficiency. The LCD-iPSCs expressed pluripotency-related genes *Oct4*, *Sox2*, *Nanog*, and *Prdm14*. Moreover, the LCD-iPSCs could differentiate into all three germ layers in vitro and form chimeras. The mesenchymal-to-epithelial transition (MET) is critical for somatic cell reprogramming. We found that the expression of mesenchymal genes (*Snail2* and *Twist*) decreased and that of epithelial genes (*DSP*, *Cldn3*, *Crb3*, and *Ocln*) dramatically increased in OR-MEF treated with LCD for 3 days, indicating that the MET process effectively occurred in LCD-treated OR-MEF. Thus, LCD could enhance the generation of iPSCs from somatic cells by promoting MET in the early stages of reprogramming.

## Materials and Methods

### Reprogramming and Cell Culture

We used reprogrammable mouse embryonic fibroblast (MEF) cells carrying Oct4-GFP and a dox-inducible reprogramming factor cassette (Oct4, Sox2, Klf4, and c-Myc). Reprogramming was induced in mouse ESC medium (DMEM; Gibco, USA) 10% fetal bovine serum (FBS; Gibco), 10% KnockOut Serum Replacement (KSR; Gibco), 1 × sodium pyruvate, 1 × non-essential amino acids, 1 × penicillin/streptomycin/glutamine (Invitrogen, USA), 1 mM β-mercaptoethanol (Gibco), and 10^3^ units/ml leukemia inhibitory factor (ESGRO/LIF; Millipore, USA) in the presence of doxycycline (1 μg/ml) and LCD. The LCD was prepared by Professor Goo Yoon according to previous reports [17]. The reprogramming efficiency was measured by counting the number of Oct4-GFP-positive colonies on days 7 and 14 after dox treatment. Only Oct4-GFP-positive colonies were selected and cultured on inactivated mouse embryonic fibroblasts (MEFs) in mouse ESC medium.

### Fluorescence-Activated Cell Analysis

Oct4-GFP ESCs were dissociated with trypsin-EDTA, washed with DMEM containing 15% FBS and resuspended with 1 × PBS containing 0.1% BSA. Large clumps of cells were removed using a cell strainer (SPL). The cells were analyzed and sorted on FACSCalibur flow cytometers (BD Biosciences, USA) at the Center for University-wide Research Facilities (CURF) at Jeonbuk National University. Data were analyzed with FACsDiva software. Three biological replicates were analyzed for each sample.

### RNA Isolation and qRT-PCR

Total RNA was isolated using the RNeasy Mini Kit (Qiagen, The Netherlands) and treated with DNase to remove genomic DNA contamination. One microgram of total RNA was reverse-transcribed using the Superscript III reverse transcriptase kit (Invitrogen) and oligo (dT) primer (Invitrogen) according to the manufacturer’s instructions. Quantitative polymerase chain reaction (PCR) reactions were set up in duplicate with the Power SYBR Green Master Mix (Takara, Japan) and analyzed with the Roche LightCycler 5480 (Roche, Switzerland). The primers for qRT‐PCR used were as follows: Oct4 sense 5′-GAAAGCAACTCAGAGGGAACCT-3′, Oct4 antisense 5′- AGGTGATCCTCTTCTGCTTCAG-3′ Oct4 (endo) sense 5′-GATGCTGTGAGCCAAGGCAAG-3′, Oct4 (endo) antisense 5′-GGCTCCTGATCAACAGCATCAC-3′, Sox2 sense 5′-CATGAGAGCAAGTACTGGCAAG-3′, Sox2 antisense 5′-CCAACGATATCAACCTGCATGG-3′, Sox2 (endo) sense 5′-GACTAGGGCTGGGAGAAA GA, Sox2 (endo) antisense 5′-CTCGGGCTCCAAACTTCTCT-3′ Nanog sense 5′-CTTTCACCTATTAAGGTGCTTGC-3′, Nanog antisense 5′-TGGCATCGGTTCATCATGGTA-3′, Prdm14 sense 5′-ACAGCCAAGCAATTTGCACTAC-3′, Prdm14 antisense 5′-TTACCTGGCATTTTCATTGCTC-3′, Snail sense 5′-TCCAAGAAGCCCAACTACAG-3′, Snail antisense 5′-GCCACTGGGTAAAGGAGAGT-3′, Twist sense 5′-GAGCAGAGACCAAATTCACAAG-3′, Twist antisense 5′-GGGACACAAACGAGTGTTCA-3′, Zep1 sense 5′-TTCTGCAGCAACAAGACACC-3′, Zep1 antisense 5′-AACTGGGAAAATGCATCTGG-3′, FN1 sense 5′-CTGAAGTCGCAAGGAAACAAG-3′, FN1 Antisense 5′-TCCTACCGTTGTAGGTGAACG-3′, Dsp sense 5′-TACCAGACCCTGGTCATTCAG-3′, Dsp antisense 5′-CCATGTCTCCTGTTTGTCGTT-3′, Cldn3 sense 5′-ACCAACTGCGTACAAGACGAG-3′, Cldn3 antisense 5′-CGGGCACCAACGGGTTATAG-3′, Crb3 sense 5′-GGACCCTTTCACAAATAGCA-3′, Crb3 antisense 5′-TGAGCAGAAACAGTCCCACT-3′, Ocln sense 5′-ACAGTCCAATGGCCTACTCCT-3′, Ocln antisense 5′-CTGCCTGAAGTCATCCACACT-3′.

### Immunocytochemistry

For immunocytochemistry, cells were fixed with 4% paraformaldehyde for 20 min at room temperature. After the cells were washed with PBS, they were treated with PBS containing 10% normal goat serum and 0.03% Triton X‐100 for 45 min at room temperature. The primary antibodies used were anti‐OCT4 [OCT4; monoclonal, 1:500, Millipore], anti‐SOX2 [SOX2; polyclonal, 1:500, Millipore], anti-tubulin, βIII [TUJ1; monoclonal, 1:1,000, Millipore], anti-alpha-SMA (α-SMA) (monoclonal, 1:500, Millipore), and anti‐SOX17 (SOX17; monoclonal, 1:200, Millipore). For the detection of primary antibodies, fluorescently labeled (Alexa Fluor 488 or 568; Molecular Probes, USA) secondary antibodies were used according to the manufacturer’s specifications.

### Western Blotting

Western blotting was used to compare the reprogramming factor (Oct4) in the control and treatment groups. Specifically, after the respective incubation times at the different temperatures, the proteins were extracted from the fibroblasts using RIPA buffer (150 mM sodium chloride, 1% Triton X-100, 0.1% SDS, 1% sodium deoxycholate, 50 mM Tris-HCl pH 7.5, and 2 mM EDTA) with protease inhibitor added.

Protein concentrations were calculated using a DC Protein Assay Kit (Bio-Rad, USA). The sample proteins (30 μg) were electrophoresed using 12% SDS PAGE gel (Bio-Rad) and transferred to nitrocellulose membranes. The membranes were blocked with 5% skim milk at room temperature and then incubated overnight at 4ºC with primary antibodies. After incubation, the membranes were washed three times for 10 min with Tris buffer solution and 0.5% Tween 20 (TBST), and then incubated with HRP-conjugated secondary antibody for 1 h at room temperature. Finally, a chemiluminescent substrate (Invitrogen) was used to visualize the protein bands and capture images on an iBright CL1000. Oct4 was detected and normalized to the β-tubulin level in each lane. The primary antibody for β-tubulin was diluted with 1:2500 mouse monoclonal IgG (Santa Cruz Biotechnology, USA), and Oct4 was diluted with 1:2500 mouse monoclonal IgG (Enzo Life Sciences, USA). The secondary antibody used was goat anti-mouse IgG (Enzo Life Sciences) diluted with 1:5000 5% skim milk.

### Chimera Formation

LCD-iPSCs were aggregated with denuded post-compacted eight-cell-stage embryos to obtain an aggregate chimera. Eight-cell embryos flushed from 2.5‐dpc B6D2F1 female mice were cultured in microdrops of embryo culture medium under mineral oil. The cells were trypsinized for 10 s and clumps of LCD-iPSCs (4–10 cells) were selected and transferred into microdrops containing zona-free, eight-cell embryos. Morula‐stage embryos aggregated with LCD-iPSCs were cultured overnight at 37°C under 5% CO_2_. The aggregated blastocysts were transferred into one uterine horn of 2.5‐dpc pseudopregnant recipients.

### DNA Extraction and Genotyping

LCD-iPSCs were aggregated and cultured with zona-free, post-compacted 8-cell-stage mouse embryos. Oct4-GFP^+^ clumps of iPSCs (4-10 cells) were selected after trypsinization and transferred into G2 drops containing zona-free, 8-cell-to-morula stage embryos. The iPSCs aggregated with embryos were cultured overnight at 37°C in 5% CO_2_. After 24 h, Oct4-GFP^+^ aggregated blastocysts were selected and transferred to the uterine horns of 2.5 dpc pseudopregnant recipient mice. To analyze chimerism, genomic DNA was isolated from the organs of a 13.5 dpc chimeric fetus. PCR amplification was performed using 100 ng of each genomic DNA for 28 cycles (94°C for 5 min, 58°C for 30 s, and 72°C for 7 min). The primers used for genotyping were as follows: EGFP sense, 5′-GCAAGCTGACCCTGAAGTTCA-3′; EGFP antisense, 5′-TCACCTTGATGCCGTTCTTCT-3′; ACTB sense, 5′-CGCCATGGATGACGATATCG-3′; and ACTB antisense, 5′-CGAAGCCGGCTTTGCACATG-3′.

### Pull-Down Assay

According to the manufacturer’s instructions, lyophilized CNBr-activated Sepharose 4B matrix powder (1 g, GE Healthcare, Swenden) was dissolved in 1 mM HCl, and then washed on a sintered glass filter for 5 min with 1 mM HCl solution. CNBr-activated Sepharose 4 B matrix beads (0.2 g) were quickly transferred into the coupling buffer, containing 0.1 M NaHCO3 (pH 8.3) and 0.5 M NaCl, and then coupled to 1 mg of LCD. The LCD-conjugated Sepharose 4B beads or control Sepharose 4B beads were incubated with cell lysates (500 μg), respectively, and then mixed overnight at 4°C. Following that, the beads were washed once with couple buffer (0.1 M NaHCO3 (pH 8.3) and 0.5 M NaCl), and dissolved into blocking buffer (0.1 M Tris-HCl (pH 8.0), then mixed overnight at 4°C. After blocking, the beads were detected by SDS-polyacrylamide gel electrophoresis and western blot analysis.

## Results

### LCD Enhances Reprogramming from Fibroblasts

**Fig. 1:**
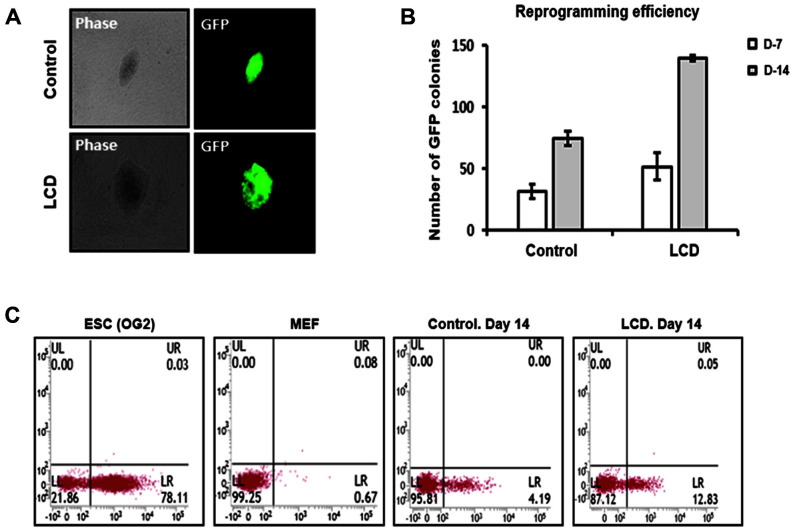
LCD enhances reprogramming from fibroblasts. (**A**) Oct4-GFP immunofluorescence of reprogrammed MEF with or without LCD. (**B**) Time-course quantification of GFP^+^ colonies in reprogrammed MEF at day 7 and day 14 with or without LCD indicated reprogramming efficiency. (**C**) FACS analysis of Oct4-GFP expression in MEFs after reprogramming at day 14 with or without LCD.

We first optimized the concentration of LCD for MEF survival. We performed the MTS assay to determine the effect of LCD on the viability of MEF cells (Fig. S1A). The MEFs were cultured in MEF medium containing LCD (0, 1, 3, 5, 10, and 20 μM) for 48 h. LCD at 1 μM concentration did not affect the viability of the MEF cells. However, their viability decreased at concentrations > 1 μM LCD. Therefore, 1 μM LCD was used for reprogramming in this study. To determine the effect of LCD on reprogramming, *Oct4*-GFP reprogrammable mouse embryonic fibroblasts were treated with doxycycline and LCD [18]. The reprogrammable MEFs were seeded in plates and cultured in doxycycline-containing mouse ESC medium, with or without 1 μM LCD, for 14 days (Fig. 1A). Reprogrammed *Oct4*-GFP colonies in mouse ESC medium with and without 1 μM LCD were observed. The number of *Oct4*-GFP colonies was then measured (Fig. 1B). The number of GFP colonies with ESC morphology increased threefold after treatment with LCD compared to that of the control. Moreover, fluorescence-activated cell sorting (FACS) analysis showed that GFP-positive cells were more abundant in reprogramming cells with LCD (Fig. 1C). Thus, these results suggest that LCD can enhance reprogramming of MEF.

### Pluripotency of the LCD-iPSCs

**Fig. 2:**
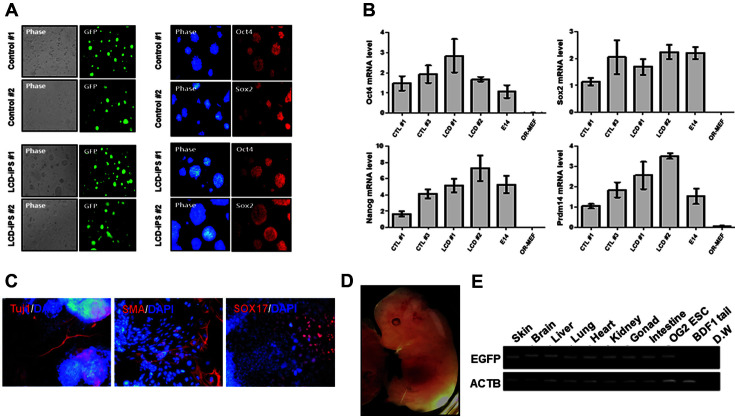
Pluripotency of the LCD-iPSCs. (**A**) Immunofluorescence staining of core pluripotency markers, Oct4 and Sox2 in LCD-iPSC like ESCs. (**B**) The qRT-PCR analysis for expression of core pluripotency markers, Oct4, Sox2, Nanog, and Prdm14 in LCD-iPSC like ESCs. (**C**) The chimeric mouse embryos from LCD-iPSCs. (**D**) Differentiation potential of LCDiPSCs to three germ layers, stained for markers of ectoderm (Tuji1), mesoderm (α-SMA), and endoderm (Sox17). (**E**) Genotyping analysis data for detection of differentiation ability of LCD-iPSC in chimeric embryo.

We attempted to pick only *Oct4*-GFP colonies of the culture control and LCD-iPSCs and established two LCD-iPSC lines (#1 and #2) (Fig. 2A). LCD-iPSCs exhibited a typical ES-like morphology and expressed *Oct4*-GFP (Fig. 2A). Immunocytochemistry showed that LCD-iPSCs expressed the core pluripotency markers *Oct4* and *Sox2* (Fig. 2A). We also confirmed the expression of *Oct4*, *Sox2*, *Nanog*, and *Prdm14* in LCD-iPSCs #1 and #2 (Fig. 2B), demonstrating that LCD-iPSCs highly express pluripotent genes in mouse ESCs. The LCD-iPSCs efficiently formed embryoid bodies (EBs), and immunostaining analysis revealed that the LCD-iPSCs differentiated into all three germ layers: ectoderm (Tuj1), mesoderm (α-SMA), and endoderm (Sox17) in vitro (Fig. 2C). To determine the in vivo differentiation of LCD-iPSCs, LCD-iPSCs were cocultured with 8 cell embryos. *Oct4*-GFP^+^ LCD-iPSCs were incorporated into the inner cell mass of blastocysts and chimeric embryos were formed (Fig. 2D). Genotyping analysis showed that the LCD-iPSCs could differentiate into skin, brain, liver, heart, kidney, gonad, and intestine in vivo (Fig. 2E). These results demonstrate that iPSCs treated with LCD maintained pluripotency in vitro, expressed pluripotent markers, and had differentiation potential in vitro and in vivo.

### LCD Promotes MET in Early Stage of Reprogramming

**Fig. 3:**
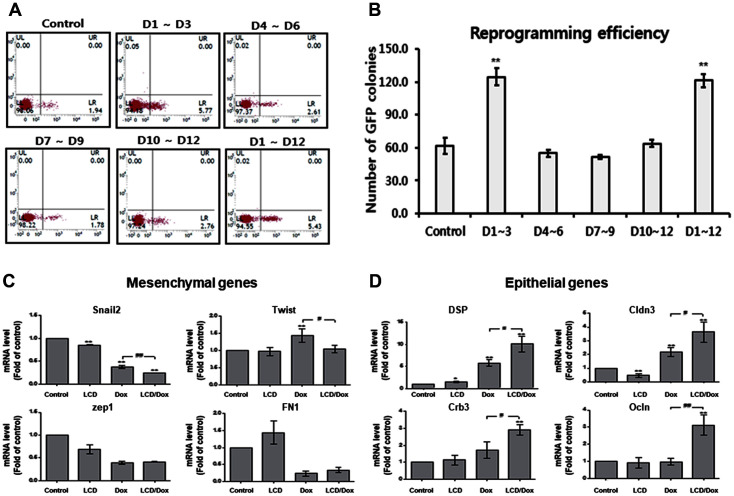
LCD promotes MET in early stage of reprogramming. (**A**) FACS analysis of Oct4-GFP positive cells in reprogramming cells with LCD (days 1–3, 4–6, 7–9, 10–12, 1–12). (**B**) Number of Oct4-GFP positive colonies in reprogramming cells with LCD (days 1–3, 4–6, 7–9, 10–12, 1–12). (**C**) The qRT-PCR analysis for the expression of mesenchymal genes (Snail2, Twist, Zep1, FN1) and (**D**) epithelial genes (DSP, Cldn3, Crb3, and Ocln).

Reprogramming of somatic cells follows a sequence of epigenetic changes similar to those undergoing transcriptional and histone modification changes prior to changes in DNA methylation during normal somatic differentiation [6, 7]. To determine the effect of LCD on the reprogramming phase, we treated OR-MEFs with LCD during reprogramming with doxycycline on days 1–3, 4–6, 7–9, 10–12, and 1–12 and counted *Oct4*-GFP^+^ colonies. Interestingly, reprogrammed cells treated with LCD on days 1–3 contained more *Oct4*-GFP^+^ colonies (> 2 fold) (Fig. 3A). However, no difference in the number of *Oct4*-GFP^+^ colonies was observed in the treatment groups on days 4–6, 7–9, 10–12, and the untreated group (control). Moreover, no increase in the number of *Oct4*-GFP^+^ colonies was observed on days 1–12 compared with that on days 1–3. These results indicate that LCD enhances reprogramming at an early stage and that continuous LCD treatment cannot improve the efficiency of reprogramming. FACS data showed that 1 ~ 2% *Oct4*-GFP^+^ cells were present in reprogramming cells without LCD, treatment with LCD on days 4 ~ 6, 7 ~ 9, 10 ~ 12, and 5% *Oct4*-GFP^+^ cells were present in reprogramming cells with LCD on days 1 ~ 3 and 1 ~ 12 (Fig. 3B). Mesenchymal-to-epithelial transition (MET) is essential for the generation of iPSCs at the early stages of reprogramming [4]. We checked the expression levels of MET-related genes in reprogramming cells with LCD on day 3. The qRT-PCR analysis showed that mRNA expression of mesenchymal genes (*Snail* and *Twist*) was downregulated and that of epithelial genes (*DSP*, *Cldn3*, *Crb3*, and *Ocln*) was upregulated in reprogramming cells treated with LCD and dox compared to that in cells treated with dox only (Figs. 3C and 3D). There was no difference in the expression levels of mesenchymal and epithelial genes in MEF-treated LCD without dox compared to control MEF, indicating that LCD could regulate the MET process by interacting with reprogramming factors. Thus, these results suggest that MET could be boosted at the early stage of reprogramming by treatment with LCD.

### LCD Regulates Stability of Reprogramming Factors

**Fig. 4:**
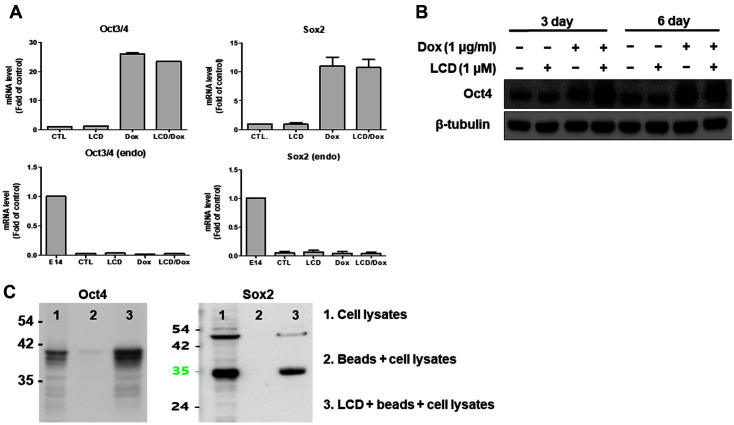
LCD regulates stability of reprogramming factors. (**A**) The qRT-PCR analysis of total or endogenous Oct4 and Sox2 expression. (**B**) Western blot analysis of OCT4 protein expression in LCD-iPSC-like ESCs (day 3, day 6). (**C**) The pulldown assay demonstrated that LCD can be directly bound to OCT4 and SOX2 proteins.

To determine whether LCD affects the expression levels of reprogramming factors in the early stage of reprogramming, we examined the expression levels of *Oct4* and *Sox2* (full and endogenous levels) in reprogramming cells on day 3 with or without LCD (Fig. 4A). The expression levels of total *Oct4* and *Sox2* in reprogramming cells with only dox were similar to those with dox and LCD (Fig. 4A). Moreover, there was no change in gene expression of endogenous *Oct4* and *Sox2* with and without LCD (Fig. 4A), indicating that LCD does not affect expression levels of endogenous genes. Next, we checked the protein levels of reprogramming factors in reprogramming MEFs on days 3 and 6 (Fig. 4B). *Oct4* protein levels in reprogramming cells with LCD were higher than those in cells without LCD on day 3. These results indicate that LCD affects the protein levels of reprogramming factors in the early stages of reprogramming. To determine whether reprogramming factors (*Oct4* and *Sox2*) might be molecular targets of LCD, we conducted pull-down experiments using mouse ESC cell line (E14) lysates and LCD-conjugated beads. The pull-down assay showed that LCD could be directly bound to *Oct4* and *Sox2* proteins (Fig. 4C). These results suggest that LCD affects the stability of reprogramming factors by binding to exogenous proteins during the early stages of reprogramming.

## Discussion

We described here that the natural compound licochalcone D (LCD) improves reprogramming efficiency. To verify the pluripotency of LCD-iPSCs, we confirmed the expression of pluripotency-related genes, three-germ layer differentiation, and chimera formation in LCD-iPSCs. Core pluripotency-related genes, such as *Oct4*, *Nanog*, *Sox2*, and *Prdm14*, were expressed in LCD-iPSCs. Furthermore, LCD-iPSCs have the ability to differentiate into all three germ layers in vitro as follows: endoderm (Sox17), mesoderm (α-SMA), and ectoderm (Tuj1), and can form chimeras. The most pivotal process in somatic cell reprogramming is the mesenchymal-to-epithelial transition (MET). In OR-MEF treated with LCD for 3 days, the expression of mesenchymal genes (*Snail2* and *Twist*) was downregulated and the expression of epithelial genes (*DSP*, *Cldn3*, *Crb3*, and *Ocln*) was upregulated. These results show that the MET process takes place rapidly for 3 days in LCD-treated OR-MEF cells. Collectively, LCD could increase the efficiency of iPSC formation from somatic cells by accelerating the MET process at an early stage of reprogramming.

During the reprogramming of fibroblasts, their transformation into tightly packed, rounded cell clusters in a MET-like process was noticeable [1, 19] , contrary to the EMT program. The MET process is facilitated during the early induction phase of reprogramming into iPSCs in vitro [19-21]. In the regulation of EMT, TGF-β signaling plays a pivotal role in transforming epithelial cells into mesenchymal cells in healthy humans and mice [21, 22]. Reprogramming can be facilitated by the activation of *Sox2*- and Myc-dependent functions through small molecules for TGF-β receptor antagonists [12, 23]. The fusion of the TGF-β inhibitor SB431542 and LDN193189 elevated the reprogramming efficiency of the MET transition [24]. Zinc ﬁnger transcription factors (Snail, Slug, Zeb1, and Zeb2) maintain mesenchymal phenotype by directly repressing epithelial gene expression [25]. TGF-β and Snail genes must be repressed by the MET process to reprogram fibroblasts into iPSCs [4]. Consistent with the acquisition of epithelial-like markers, these transcription factors were suppressed in parallel with the loss of ﬁbroblast markers Cdh2 and Thy1 in reprogramming cultures. Jose *et al*. showed that major gene expression changes occur in two discernible phases during iPSC formation and refractory Thy1-positive cells in reprogramming cells could not undergo MET [7]. They also detected increased OCT4 protein levels in SSEA1-positive cells than in Thy1-positive cells (day 3), and furthermore, refractory Thy1-positive cells could be rescued by elevated OKSM protein levels [7]. These ﬁndings indicate that initiation of MEF reprogramming is related to protein levels of OKSM and MET [26]. We showed that there was no significant difference in the expression levels of transgenes (*Oct4* and *Sox2*) in reprogramming cells with only dox and LCD (Fig. 4A). We detected increased *Oct4* protein levels in reprogramming cells with dox and LCD on day 3 (Fig. 4B). These data suggest that LCD enhances the MET process in reprogramming cells by elevating the level of OCT4 protein.

Existing studies are inefficient in generating iPSCs while being accompanied by significant complexity and difficulty in mechanical research and high-throughput screening. The efficiency of alkaline phosphatase-positive (AP+) colony formation using four Yamanaka factors (Sox2, Klf4, Oct4, c-Myc; SKOM) in mouse fibroblasts is approximately 1% of the total population [27]. Recently, reprogramming reports demonstrated that reprogramming efficiency increased by treatment with small molecules, which regulate epigenetic changes. In MA Esteban *et al*., the addition of vitamin C, which is readily available in the diet as a natural compound, increased the reprogramming efficiency of somatic cells and significantly increased iPSC colonization in mice and humans [13]. Notably, vitamin C was more effective than the histone deacetylase inhibitor valproic acid (VPA) in increasing GFP^+^ cells in SKO-infected MEFs [11]. There is considerable interest in finding natural compounds that increase the efficiency of reprogramming without increasing the risk of mutations for reprogramming and side effects. Natural compounds are defined as being produced by biological sources or living organisms [28]. In general, herbal remedies or plant extracts have been prescribed as natural compounds to treat various ailments in most countries in the field of medicine [29].

We showed that licochalcone D (LCD), a flavonoid natural compound mainly existing in the root of *Glycyrrhiza inflata*, increases the reprogramming efficiency of MEF to generate pluripotent iPSCs. NF-κB plays an essential role in maintaining the mesenchymal state after EMT, and NF-κB plays an essential role in the induction of EMT in Ras-transfected mammary epithelial cells, as inhibition causes reversal of EMT [16]. IKK activity is involved in NF-κB stimulation of transcriptional activity by Akt, [30-32] and other researchers have suggested that PI3K or Akt may also stimulate NF-κB activity through targeting the p65 subunit of NF-κB20 [21]. NF-κB p65 is involved in epithelial cells [15, 33, 34]. Licochalcone D (LCD) is used as an inhibitor that significantly inhibits LPS-induced NF-kB transcriptional activation by abrogating the phosphorylation of NF-kB p65 at serine 276. Through this mechanism, LCD efficiently controlled MET in the process of reprogramming somatic cells into iPSCs. However, it is unclear how LCD directly regulates and engages the MET process. Therefore, further mechanistic research is required.

This study will help to identify other natural compounds that are efficient in reprogramming somatic cells, and also to understand and establish mechanisms. Furthermore, it could help to develop new methods for regenerative medicine.

## Supplemental Materials

Supplementary data for this paper are available on-line only at http://jmb.or.kr.
