# Erratum to: Acceleration of Mesenchymal-to-Epithelial Transition (MET) during Direct Reprogramming Using Natural Compounds

**DOI:** 10.4014/jmb.2022.3212.1632

**Published:** 2022-12-28

**Authors:** Ji-Hye Seo, Si Won Jang, Young-Joo Jeon, So Young Eun, Yean Ju Hong, Jeong Tae Do, Jung-il Chae, Hyun Woo Choi

**Affiliations:** 1 Department of Dental Pharmacology, School of Dentistry, Jeonbuk National University, Jeonju 54896, Republic of Korea; 2 Department of Agricultural Convergence Technology, Jeonbuk National University, Jeonju 54896, Republic of Korea; 3 Disease Target Structure Research Center, Korea Research Institute of Bioscience and Biotechnology (KRIBB), Daejeon 34141, Republic of Korea; 4 Musculoskeletal and Immune Disease Research Institute School of Medicine, Wonkwang University, Iksan 54538, Republic of Korea; 5 Department of Psychiatry and Molecular Neurobiology Laboratory, McLean Hospital and Program in Neuroscience, Harvard Medical School, Belmont, MA 02478, USA; 6 Department of Stem Cell and Regenerative Biotechnology, KU Institute of Science and Technology, Konkuk University, Seoul 05029, Republic of Korea; 7 Department of Animal Science, Jeonbuk National University, Jeonju 54896, Republic of Korea

In the article titled Acceleration of Mesenchymal-to-Epithelial Transition (MET) during Direct Reprogramming Using Natural Compounds, the funding acknowledgment was partially incorrect as published.

Original version

Acknowledgments

This research was supported by the National Research Foundation of Korea (NRF) funded by the Ministry of Education (NRF-2021R111A1A01048981) and the Korea government (MSIP; Ministry of Science, ICT & Future Planning) (NRF-2017R1C1B5077043).

**Corrected version**

**Acknowledgments**

This research was supported by the National Research Foundation of Korea (NRF) funded by the Ministry of Education (NRF-2021R111A1A01048981) and the National Research Foundation (NRF) grant funded by the Korea government (MSIT) (NRF-2020R1C1C1014148).

