# Supplementary material for: Acceleration of Mesenchymal-to-Epithelial Transition (MET) during Direct Reprogramming Using Natural Compounds
Source: J Microbiol Biotechnol. 2022 Sep 19;32(10):1245–52. doi: 10.4014/jmb.2208.08042 (PMC9668095; doi:10.4014/jmb.2208.08042)
Supplement: Supplementary file 1 [file jmb-32-10-1245-supple.pdf]

J. Microbiol. Biotechnol.
https://doi.org/10.4014/jmb.2208.08042

Supplementary Materials

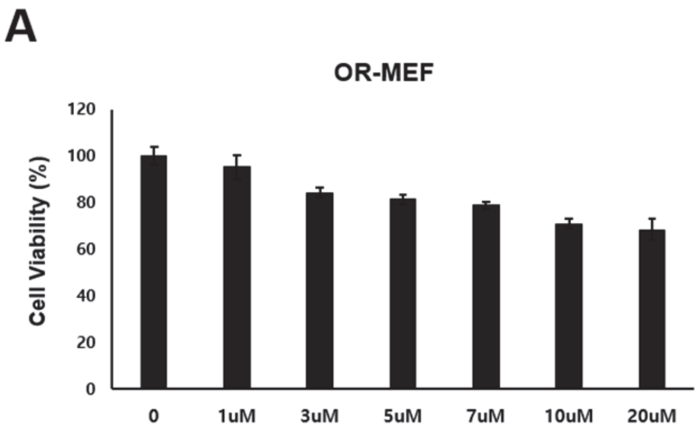

Supplementary Fig. 1. MTS assay for effect of LCD on the viability

(**A**) The MEF were cultured in different concentration of LCD for 48hr.
